# Dose-Dependent Microglial and Astrocytic Responses Associated With Post-ischemic Neuroprotection After Lipopolysaccharide-Induced Sepsis-Like State in Mice

**DOI:** 10.3389/fncel.2020.00026

**Published:** 2020-02-12

**Authors:** Maryam Sardari, Egor Dzyubenko, Ben Schmermund, Dongpei Yin, Yachao Qi, Christoph Kleinschnitz, Dirk M. Hermann

**Affiliations:** Department of Neurology, University Hospital Essen, Essen, Germany

**Keywords:** microglial activation, middle cerebral artery occlusion, neuroprotection, morphological analysis, reactive astrocyte, sepsis

## Abstract

In contrast to lipopolysaccharide (LPS)-induced preconditioning, which has repeatedly been examined in the past, the effects of post-ischemic LPS-induced sepsis, although clinically considerably more important, have not systemically been studied. We exposed mice to transient intraluminal middle cerebral artery occlusion (MCAO) and examined the effects of intraperitoneal LPS (0.1 or 1 mg/kg) which was administered 24 h post-ischemia. Post-ischemic glial reactivity, neuronal survival and neurological outcome were differently modulated by the higher and the lower LPS dose. Although both doses promoted neuronal survival after 72 h, the underlying mechanisms were not similar. Mice receiving 1 mg/kg LPS exhibited transient hypothermia at 1 and 3 hours post sepsis (hps), followed by reduced focal neurological deficits at 24, 48 and 72 hps. The lower dose (0.1 mg/kg) did not induce hypothermia, but reduced microglia/macrophage activation with the appearance of an anti-inflammatory CD206 positive cell phenotype in the brain parenchyma. Together, our results indicate a novel, dose-dependent modulation of microglial cells that is intricately involved in brain protection.

## Introduction

Lipopolysaccharide (LPS) is a major component of the outer membrane of gram-negative bacteria, such as *Escherichia coli*. LPS activates pro-inflammatory signals through toll-like receptor-4 (TLR4), which in the brain is mainly expressed by microglial cells (Qin et al., 2005), the central element of the brain’s innate immune system that provides first-line defense against inflammatory stimuli (Ransohoff and Brown, 2012). Systemic LPS administration can exhibit varying effects in different animal models. LPS delivery induces a febrile response in rats and guinea pigs (Sehic and Blatteis, 1996; Bao et al., 2018) but not in mice. Thus, LPS is considered an attractive model of gram-negative sepsis-like states. In rats, post-ischemic LPS delivery exacerbated systemic inflammatory responses, increased infarct volume and increased neurological deficits (Yousuf et al., 2013). In mouse models of middle cerebral artery occlusion (MCAO), LPS delivery before ischemia confered protection *via* inflammatory pre-conditioning in rats and mice (Bastide et al., 2003; Rosenzweig et al., 2004). In mice, intravenous LPS administration at 2 h after cytokine-induced brain injury gave rise to the neuroprotection and reduced the number of neutrophils and macrophages infiltrating into the brain (Davis et al., 2005). In mixed neuronal cultures, LPS increased neuronal survival and anti-inflammatory IL-10 production when administered after scratch model of mechanical cell injury (Bingham et al., 2011). The combined evidence of these studies suggests the timing and dosing of LPS decisively influence effects in the ischemic brain. To elucidate the effects of post-ischemic LPS administration, we herein exposed mice to transient intraluminal MCAO, evaluating the effects of two LPS doses, 0.1 mg/kg and 1 mg/kg, on ischemic injury, astrocytic and microglial responses.

### Experimental Procedures and Neurological Tests

Experiments were conducted with government approval according to E.U. guidelines (EU Directive 2010/63) for the care and use of laboratory animals. Animals were housed in groups in an ordinary 12 h:12 h light/dark cycle. Animals were randomly attributed to treatment paradigms, and experimenters were blinded at all stages of interventions and data analysis. Male C57BL/6 mice (23–27 g, Harlan, Horst, The Netherlands) were exposed to 20 min left-sided intraluminal MCAO or sham surgery during 1.5% isoflurane anesthesia (30% O_2_, remainder N_2_O). Prior to surgery, animals received intraperitoneal injections of the analgesic buprenorphine (0.1 mg/kg; Reckitt Benckiser, Slough, UK). During the surgery, rectal temperature was kept at 37.0°C using a feedback-controlled heating system. Laser Doppler flow (LDF) was monitored by a flexible probe above the core of the middle cerebral artery territory. A midline neck incision was made. The left common and external carotid arteries were isolated and ligated, and the internal carotid artery was temporarily clipped. A silicon-coated nylon monofilament (0.21 mm tip diameter; Doccol, Sharon, MA, USA) was introduced through a small incision of the common carotid artery and advanced to the circle of Willis for MCAO. Reperfusion was initiated by monofilament removal. For pain relief, animals received daily injections of carprofen (4 mg/kg, intraperitoneally; Bayer Vital, Leverkusen, Germany) during the first 3 days post-stroke. Twenty-four hours after reperfusion, animals were treated with vehicle (normal saline) or LPS (0.1 mg/kg or 1 mg/kg intraperitoneally; from *E. coli* 0111:B4; Sigma, Deisenhofen, Germany). In parallel pre-conditioning experiments, animals were treated with vehicle (normal saline) or LPS (1 mg/kg) 24 h before MCAO surgery (Supplementary Figure S1A). Throughout the study, we used *E. coli* LPS 0111:B4 which one the most potent serotypes (Watanabe and Jaffe, 1993) and was previously shown to induce innate immune cell activation, blood-brain barrier disturbance and elevation of cytokine levels in a variety of animal models (Batista et al., 2019). Rectal temperature was monitored every 3 h from 0 to 12 hours post sepsis (hps). The rectal probes were lubricated with silicon oil and carefully inserted, taking care that no intestine injury was induced. Afterward, rectal temperature was measured every 24 h from at 24, 48 and 72 hps. Bodyweight and neurological deficits were evaluated using Clark’s neurological score (Clark et al., 1997) every 24 h from 0 to 72 hps. Animals were sacrificed 3 days post-sepsis (post-ischemic LPS delivery studies) or 3 days after MCAO (LPS pre-conditioning studies) by transcardiac perfusion with 0.1 M phosphate buffer saline (PBS) followed by 4% paraformaldehyde in PBS. Sham-operated mice were prepared by exposing mice to 1.5% isoflurane anesthesia (30% O_2_, remainder N_2_O). In these animals, a midline neck incision was made, and the left-sided carotid arteries were isolated but left intact. Brains were cut into 20 μm coronal cryostat sections.

### Volumetry/Planimetry

Brain sections collected at millimeter intervals across the brain were stained with cresyl violet. Brain volume was determined as described (Wang et al., 2018).

### Immunohistochemistry

The sections obtained from the Bregma level, i.e., the core of the middle cerebral artery territory, were stained with chicken anti-neuronal nuclei (NeuN; 1:300; ABN91, Merck-Millipore, Darmstadt, Germany), rabbit anti-dopamine and cAMP-regulated neuronal phosphoprotein (Darpp-32; 1:300; MA5-14968, Thermo Fisher Scientific, Waltham, MA, USA), rabbit anti-ionized calcium-binding adaptor protein (Iba-1; 1:500; 019-19741, Fujifilm Wako-Chemicals, Neuss, Germany) and mouse anti-glial fibrillary acidic protein (GFAP) conjugated to Alexa Fluor-555 (1:300; 3656s, Cell Signaling Technology, Frankfurt, Germany). Non-labeled primary antibodies were detected by secondary Alexa Fluor-488 or Alexa Fluor-647 labeled antibodies. Nuclei were counterstained with Hoechst-33342.

### Confocal Microscopy and Conventional Tissue Analysis

The density of NeuN+ neurons was evaluated using a Zeiss AxioObserver.Z1 inverted epifluorescence microscope using a 10× Plan-Apochromat objective. The images were pre-processed and analyzed by an open-source ImageJ (National Institutes of Health, Bethesda, MD, USA) script and the pixel classification was performed using the interactive learning and segmentation toolkit Ilastik (University of Heidelberg, Heidelberg, Germany). The area of NeuN labeling was measured in the striatum. Similarly, the survival of middle-sized dopamine-responsive neurons was quantified as the area of Darpp-32 staining. The intensity of GFAP immunolabeling was separately analyzed in the entire ipsilesional and contralesional hemispheres. For this, GFAP immunoreactivity was measured as average pixel intensity (gray value on the 16-bit scale), no background corrections were performed. The density of Iba1+ cells was analyzed in 425 × 425 × 10 μm regions of interest randomly positioned in the ipsilesional and contralesional cortex and striatum using the Carl Zeiss LSM770 confocal microscope. Six ROIs per animal per area were examined, of which mean values were formed. According to their morphology, microglia were manually classified by a trained histologist into resting (highly ramified) and activated (amoeboid shape) cells. Additionally, the density of CD206+ cells was quantified in the same ROIs following immunolabeling with primary rat anti-CD206 antibody (1:250; MA5-16871, Thermo Fisher Scientific, Waltham, MA, USA) and appropriate Alexa Fluor-647 conjugated secondary antibody.

### Statistical Analysis

For LDF, rectal temperature, body weight, focal deficits and neurological score, statistical analyses were performed using a one-way analysis of variance (ANOVA) for different time points. *Post hoc* comparison of means was carried out with the LSD test for multiple comparisons, when appropriate. For neuronal survival and inflammatory activation of glial cells, the nonparametric Kruskal–Wallis test was used. Multiple comparisons were adjusted using Bonferroni corrections. *P-values* < 0.05 were defined to indicate statistical significance. Calculations were performed using SPSS and Origin Pro 2019b statistics packages.

## Results

### Post-ischemic LPS Delivery Dose-Dependently Induces Transient Hypothermia and Neurological Recovery

**Figure 1:**
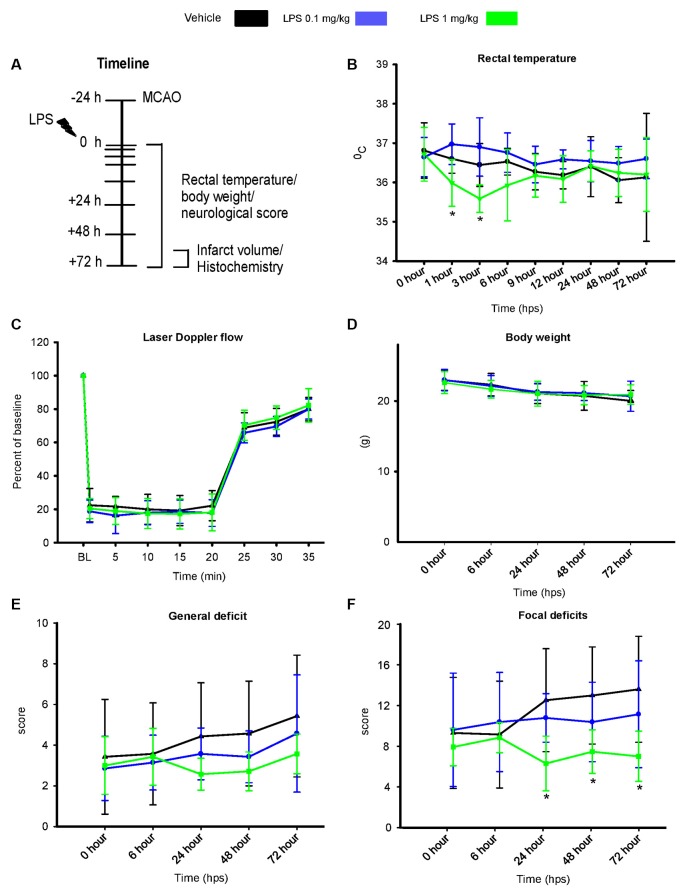
Post-ischemic lipopolysaccharide (LPS) delivery dose-dependently induces transient hypothermia and promotes neurological recovery. **(A)** Timeline of the experimental procedure, **(B)** rectal temperature, **(C)** laser doppler flow (LDF) recordings above the middle cerebral artery territory, **(D)** body weight as well as **(E,F)** general and focal neurological deficits assessed by Clark score in mice exposed to transient intraluminal middle cerebral artery occlusion (MCAO). Vehicle or LPS (0.1 or 1 mg/kg) was intraperitoneally administered at 24 h after MCAO. Note that the higher dose of LPS induces transient hypothermia at 1 and 3 hours post-sepsis (hps) and a reduction of focal neurological deficits at 24–72 hps. Results are means ± SD values. *p < 0.05 compared with vehicle group (*n* = 7 animals/group).

Two dosages of LPS (0.1 mg/kg and 1 mg/kg) were intraperitoneally administered 24 h after MCAO, according to the timeline shown in Figure 1A. Rectal temperature measurements (Figure 1B) revealed that post-ischemic delivery of 1 mg/kg, but not 0.1 mg/kg LPS led to transient hypothermia in 1 hps (*F*_(2,20)_ = 6.9, *p* < 0.05) and 3 hps (*F*_(2,20)_ = 9.6, *p* < 0.05), compared with control mice. In comparison, pre-conditioning with 1 mg/kg LPS did not significantly affect body temperature (Supplementary Figure S1). Following post-ischemic LPS delivery, body weight (Figure 1C) and LDF above the core of the middle cerebral artery territory (Figure 1D) did not differ between groups. Although we observed no significant changes in the general neurological deficit score (Figure 1E), the higher dose of LPS (1 mg/kg) reduced focal deficits starting at 24 hps (*F*_(2,20)_ = 3.8, *p* < 0.05; Figure 1F). This protective effect was maintained at later time points at 48 hps (*F*_(2,20)_ = 3.75, *p* < 0.05) and 72 hps (*F*_(2,20)_ = 3.83, *p* < 0.05; Figure 1F).

### Post-ischemic LPS Dose-Independently Reduces Infarct Volume and Increases Neuronal Survival

**Figure 2:**
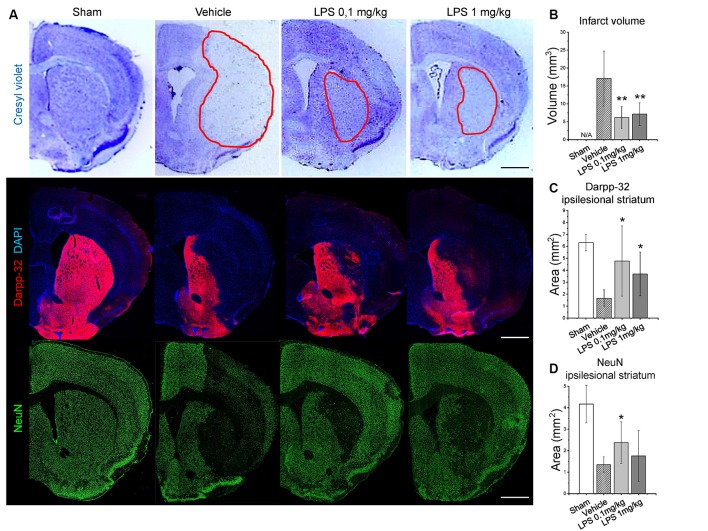
Post-ischemic LPS delivery dose-independently reduces infarct volume and increases neuronal survival. Panel **(A)** shows representative cresyl violet (infarcted tissue outlined), Darpp-32 and neuronal nuclei (NeuN) staining in the ipsilesional striatum of mice exposed to sham surgery or intraluminal MCAO which were intraperitoneally treated with vehicle or LPS (0.1 or 1 mg/kg) at 24 h after MCAO. Quantifications of infarct volume **(B)**, Darpp-32+ neurons in the ipsilesional striatum **(C)** and NeuN+ neurons **(D)** are provided. Data are means ± SD values. *p < 0.05/**p < 0.01 compared with vehicle group (*n* = 6 animals/group). Scale bars, 1 mm.

Furthermore, we performed Nissl staining (cresyl violet), Darpp-32 and NeuN immunohistochemistry to analyze infarct volume and survival of striatal neurons. For post-ischemic LPS delivery, infarct volume measurements revealed a significant neuroprotective effect of both LPS dosages (Figure 2A). Consistently, both LPS dosages increased neuronal survival, as indicated by the increased area of Darpp-32 (middle-sized dopamine-responsive neuronal marker) and NeuN (pan-neuronal marker) immunolabeling (Figures 2B–D). Similarly, pre-conditioning with 1 mg/kg LPS reduced infarct volume and increased neuronal survival (Supplementary Figure S2).

### Post-ischemic Delivery of Low Dose LPS Increases Astrogliosis and Attenuates Microglia/Macrophage Activation

**Figure 3:**
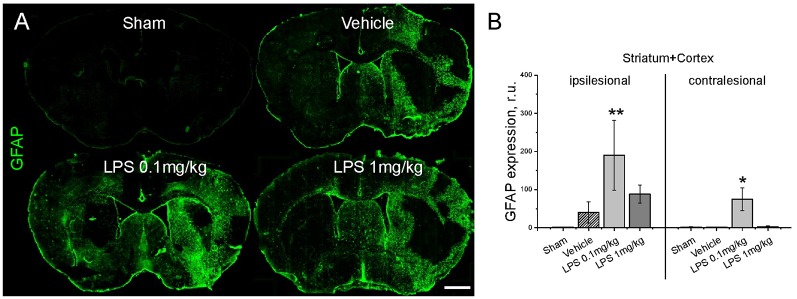
Post-ischemic delivery of low dose LPS augments astrogliosis. Panel **(A)** shows representative immunostainings of glial fibrillary acidic protein (GFAP)+ reactive astrocytes. GFAP immunoreactivity is quantified in **(B)** in the entire ipsilesional and contralesional hemispheres (striatum plus cortex) of mice exposed to sham surgery or transient intraluminal MCAO. Vehicle or LPS (0.1 or 1 mg/kg) was intraperitoneally administered at 24 h after MCAO. Data are means ± SD values. *p < 0.05/**p < 0.01 compared with vehicle group (*n* = 6 animals/group). Scale bars, 1 mm.

**Figure 4:**
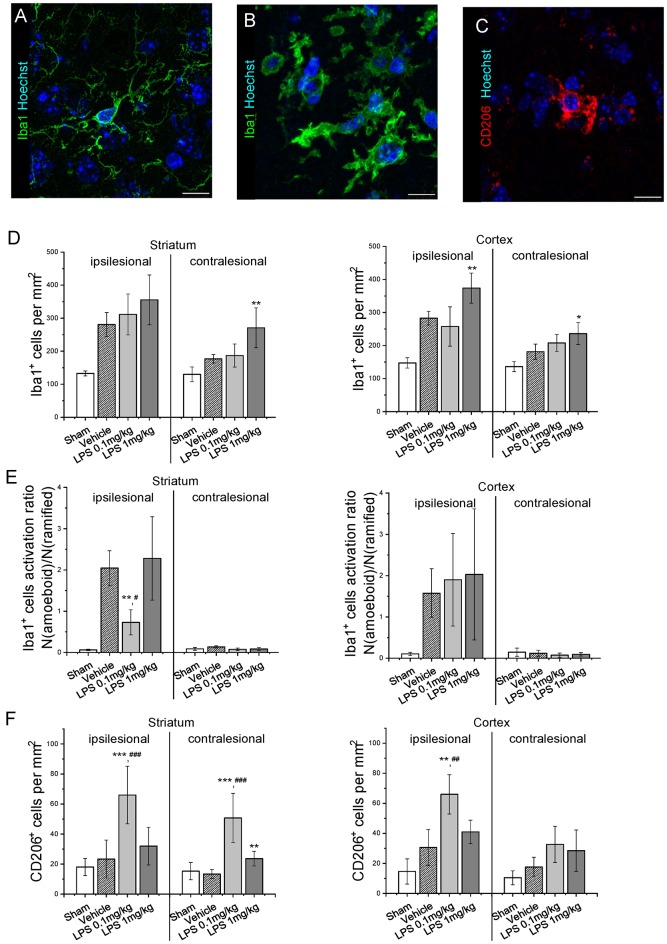
Post-ischemic low dose LPS administration attenuates microglia/macrophage activation. Panels **(A,B)** show representative ramified and amoeboid Iba1+ microglia/macrophages, **(C)** representative anti-inflammatory CD206+ microglia/macrophages. Iba1+ cell density **(D)**, Iba1+ cell activation index **(E)** and CD206+ cell density **(F)** were quantified in striatum and cortex of mice exposed to sham surgery or transient intraluminal MCAO. Vehicle or LPS (0.1 or 1 mg/kg) were intraperitoneally administered at 24 h after MCAO. Data are means ± SD values. *p < 0.05/*p < 0.01/*p < 0.001 compared with vehicle group, ^#^*p* < 0.05/^##^*p* < 0.01/^###^*p* < 0.001 compared with LPS 1 mg/kg (*n* = 6 animals/group). Scale bars, 10 μm.

Quantification of GFAP immunoreactivity (Figure 3) indicated that post-ischemic delivery of 0.1 mg/kg, but not 1 mg/kg LPS induced a significantly higher level of astrogliosis compared with the vehicle-treated group. After pre-conditioning with 1 mg/kg LPS, GFAP reactivity was not different from vehicle (Supplementary Figure S3). The different patterns of astrocyte reactivity following the post-ischemic delivery of a low and high LPS dose motivated us to study microglia/macrophage activation. Under our experimental conditions, the intricate morphology of both resting (Figure 4A) and activated (Figure 4B) cells could be visualized. Resting microglia was defined by the characteristic ramified shape, while activated microglia/macrophages were distinguished by their amoeboid morphology. The microglia/macrophage activation index was derived as the ratio between the numbers of amoeboid to ramified cells. Additionally, alternatively activated, anti-inflammatory microglia/macrophages were detected using CD206 immunolabeling (Figure 4C). As expected, Iba1+ microglia/macrophages were found in the ipsilesional striatum and cortex at 72 hps in both vehicle-treated and LPS treated groups (Figure 4D). Compared with vehicle, 1 mg/kg LPS significantly increased Iba1+ cell density in regions distant from the lesion core, i.e., contralesional striatum and ipsilesional and contralesional cortex. Compared with the vehicle and 1 mg/kg LPS, 0.1 mg/kg LPS significantly reduced microglia/macrophage activation and increased the density of CD206+ cells in the ipsilesional striatum (Figures 4E,F). Pre-conditioning with 1 mg/kg LPS reduced both density and activation index of Iba1+ microglia/macrophages in ipsilesional striatum, compared with vehicle (Supplementary Figure S4).

## Discussion

While the post-ischemic intraperitoneal delivery of 1 mg/kg, but not 0.1 mg/kg LPS induced transient hypothermia and reduced focal neurological deficits, both doses decreased infarct volume and increased neuronal survival at 72 hps. However, astrocytic reactivity was increased and microglia/macrophage activation was reduced by 0.1 mg/kg, but not 1 mg/kg LPS. Therefore, systemic immune activation post-stroke can modulate neuroinflammation in a hypothermia-independent manner and neuroprotective mechanisms induced by the higher and lower LPS doses are different. In contrast to the low dose, neuroprotective effects of the higher dose of LPS can be largely explained by the impact of hypothermia. Previously, the post-ischemic intravenous delivery of 2 mg/kg LPS was shown to reduce infarct volume and the production of reactive oxygen species (Han et al., 2002). Following the lower LPS dose, the reduced activation of microglia/macrophages and the increased density of anti-inflammatory CD206+ cells were not associated with hypothermia in our study. Interestingly, these results resembled the effect of inflammatory pre-conditioning using intraperitoneal delivery of 1 mg/kg LPS 24 h prior to MCAO surgery. In ischemic stroke, pre-conditioning with LPS reduces infarct volume, decreases the infiltration of peripheral immune cells into the brain parenchyma, attenuates microglia reactivity and increases the expression of anti-inflammatory cytokines *via* TLR4 signaling (Vartanian et al., 2011). Potentially, a similar mechanism may be recruited after post-ischemic LPS delivery.

Although an extensive analysis of cytokine expression and specific M1/M2 microglia activation markers was beyond the scope of this study, we used an alternative morphology-based approach to quantify microglia activation. Flow cytometry and cytokine measurements are valuable for evaluating neuroinflammatory responses but lack spatial information. In the context of focal cerebral ischemia, it is essential to know the spatial pattern of glial reactivity because it can affect post-stroke neural plasticity in regions which are distant from the stroke lesion (Hermann and Chopp, 2012; Sanchez-Mendoza and Hermann, 2016; Ma et al., 2017). Our approach allows comparing microglia activation in the lesion core with the adjacent and distant perilesional areas. Hereby, we attributed the reduced activation of Iba1+ cells after post-ischemic delivery of 0.1 mg/kg LPS specifically to ipsilesional striatum, while the density of CD206+ cells was increased in both ipsi- and contralesional striatum, as well as in ipsilesional cortex.

In line with the recently discovered cross-talk between microglia and astrocytes during neuroinflammation (Rothhammer et al., 2018; Vainchtein et al., 2018; Wheeler et al., 2019), we observed that LPS injection increased astroglial GFAP reactivity. Based on the detrimental outcome of impaired astrogliosis on axon regeneration (Anderson et al., 2016), this elevated astrocytic response may contribute to restorative mechanisms that support neurological recovery. Taken together, the available evidence suggests that the attenuated activation of microglia/macrophages, enrollment of CD206+ cells and enhanced astrogliosis are interacting components underlying the neuroprotective effect of LPS in ischemic stroke. Although the neuroprotective mechanisms induced by LPS are not fully resolved, the available data indicate a novel, dose-dependent mode of interaction between peripheral and brain-intrinsic immune responses that confers neuroprotection after stroke.

## Data Availability Statement

The raw data supporting the conclusions of this article will be made available by the authors, without undue reservation, to any qualified researcher.

## Ethics Statement

The animal study was reviewed and approved by Bezirksregierung North-Rhine-Westphalia.

## Author Contributions

MS and ED contributed equally to this study. MS, ED, and DH designed the study. MS, BS, and YQ performed the animal experiments. MS, BS, DY, and ED conducted histochemical staining and analyses. MS, ED, and DH analyzed the data. MS, ED, and DH drafted the manuscript. All authors finalized it.

## Conflict of Interest

The authors declare that the research was conducted in the absence of any commercial or financial relationships that could be construed as a potential conflict of interest.

## Supplementary Material

FIGURE S1 Pre-conditioning by LPS did not significantly influence body temperature. **(A)** Timeline of the experimental procedure, **(B)** rectal temperature, **(C)** LDF recordings above the middle cerebral artery territory assessed in mice exposed to transient intraluminal MCAO. Vehicle or LPS (1 mg/kg) was intraperitoneally administered at 24 h prior to MCAO. No differences were noted between the groups. Results are means ± SD values (*n* = 4 animals/group).

FIGURE S2 LPS preconditioning reduces infarct volume and increases neuronal survival in the ischemic brain. Panel **(A)** shows representative cresyl violet (infarcted tissue outlined), Darpp-32 and NeuN staining in the ipsilesional striatum of mice exposed to sham surgery or intraluminal MCAO which were treated with vehicle or LPS (0.1 or 1 mg/kg) at 24 h after MCAO. Quantifications of infarct volume **(B)**, Darpp-32+ neurons in the ipsilesional striatum **(C)** and NeuN+ neurons **(D)** are provided. Data are means ± SD values. ***p* < 0.01 compared with vehicle group (*n* = 4 animals/group). Scale bars, 1 mm.

FIGURE S3 LPS pre-conditioning does not influence the post-ischemic astrogliosis. Panel **(A)** shows representative immunostainings of GFAP+ reactive astrocytes. GFAP immunoreactivity was quantified **(B)** in the entire ipsilesional and contralesional hemispheres (striatum plus cortex) of mice exposed to sham surgery or transient intraluminal MCAO. Vehicle or LPS (1 mg/kg) was intraperitoneally administered at 24 h prior to MCAO. No differences were noted between the groups. Data are means ± SD values (*n* = 4 animals/group). Scale bars, 1 mm.

FIGURE S4 LPS preconditioning attenuates post-ischemic microglia/macrophage activation. The density of Iba1+ microglia/macrophages **(A)** and Iba1+ cell activation index **(B)** were quantified in striatum and cortex of mice exposed to sham surgery or transient intraluminal MCAO. Vehicle or LPS (1 mg/kg) was intraperitoneally administered at 24 h prior to MCAO. Data are means ± SD values. **p* < 0.05 compared with vehicle group (*n* = 4 animals/group). Scale bars, 10 μm.
