# Supplementary figures and images for: Dose-Dependent Microglial and Astrocytic Responses Associated With Post-ischemic Neuroprotection After Lipopolysaccharide-Induced Sepsis-Like State in Mice
Source: Front Cell Neurosci. 2020 Feb 12;14:26. doi: 10.3389/fncel.2020.00026 (PMC7029732; doi:10.3389/fncel.2020.00026)

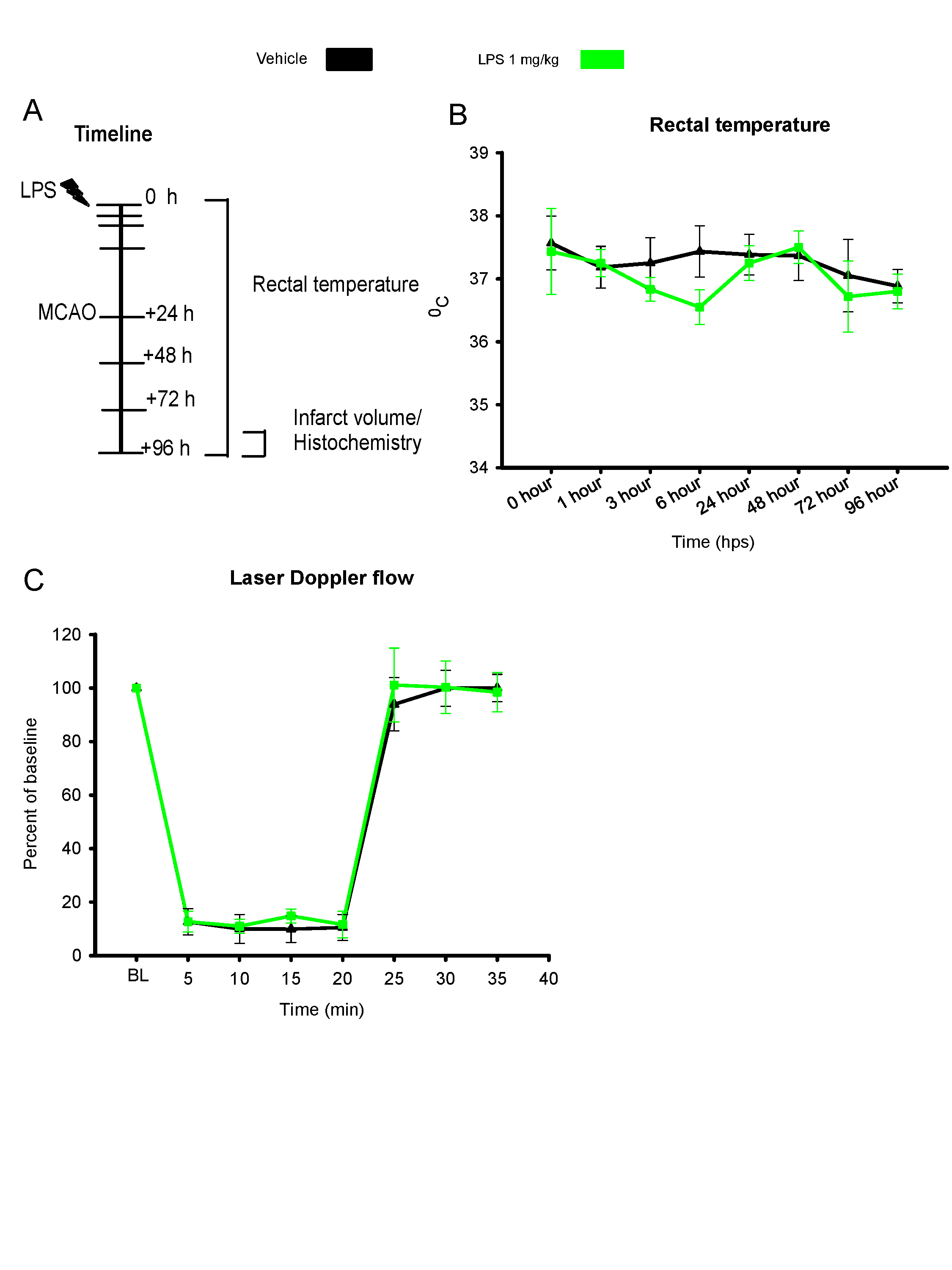

Supplement: FIGURE S1 — Pre-conditioning by LPS did not significantly influence body temperature. (A) Timeline of the experimental procedure, (B) rectal temperature, (C) LDF recordings above the middle cerebral artery territory assessed in mice exposed to transient intraluminal MCAO. Vehicle or LPS (1 mg/kg) was intraperitoneally administered at 24 h prior to MCAO. No differences were noted between the groups. Results are means ± SD values (n = 4 animals/group). [file Image_1.tiff]

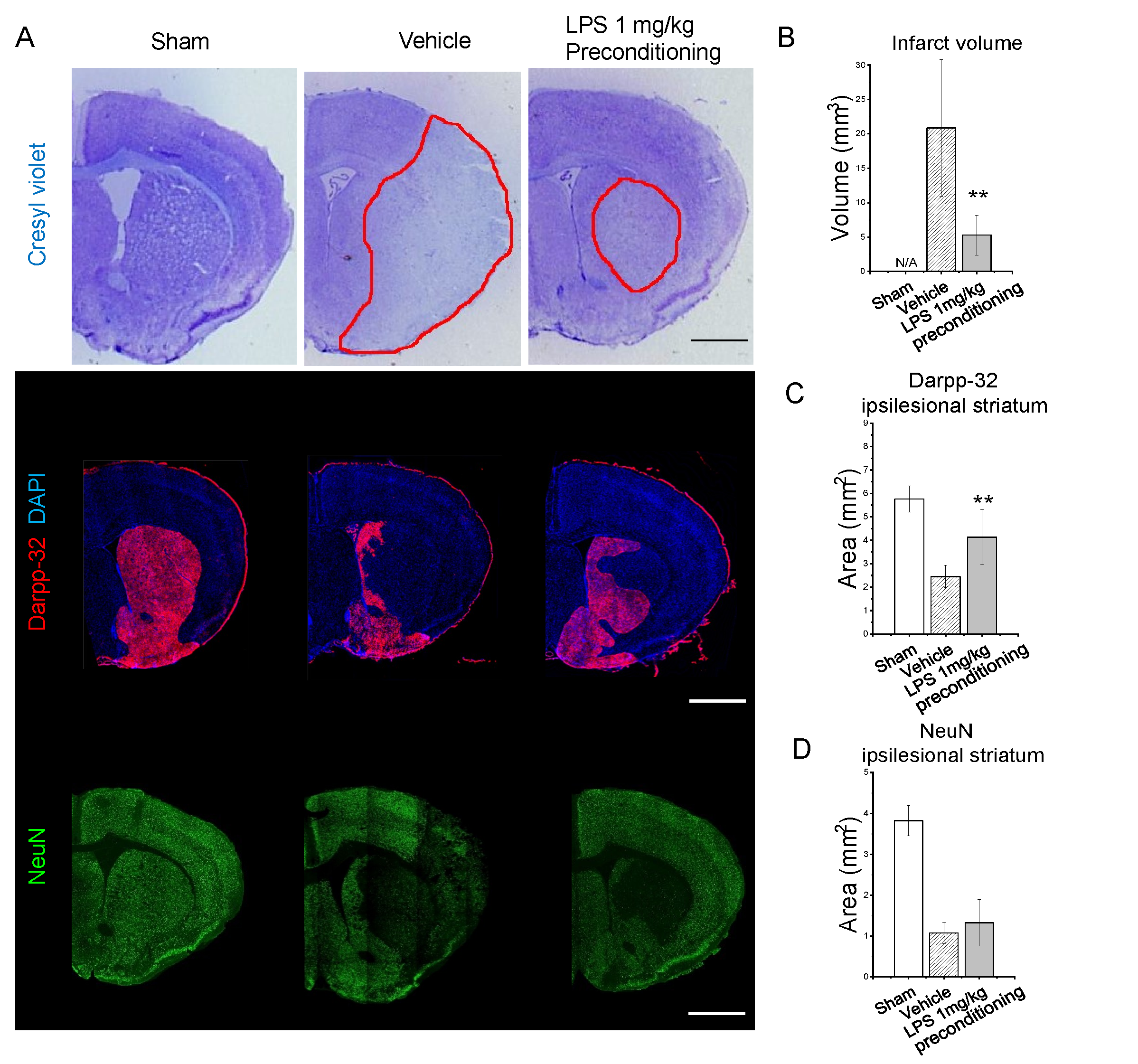

Supplement: FIGURE S2 — LPS preconditioning reduces infarct volume and increases neuronal survival in the ischemic brain. Panel (A) shows representative cresyl violet (infarcted tissue outlined), Darpp-32 and NeuN staining in the ipsilesional striatum of mice exposed to sham surgery or intraluminal MCAO which were treated with vehicle or LPS (0.1 or 1 mg/kg) at 24 h after MCAO. Quantifications of infarct volume (B), Darpp-32+ neurons in the ipsilesional striatum (C) and NeuN+ neurons (D) are provided. Data are means ± SD values. **p < 0.01 compared with vehicle group (n = 4 animals/group). Scale bars, 1 mm. [file Image_2.tiff]

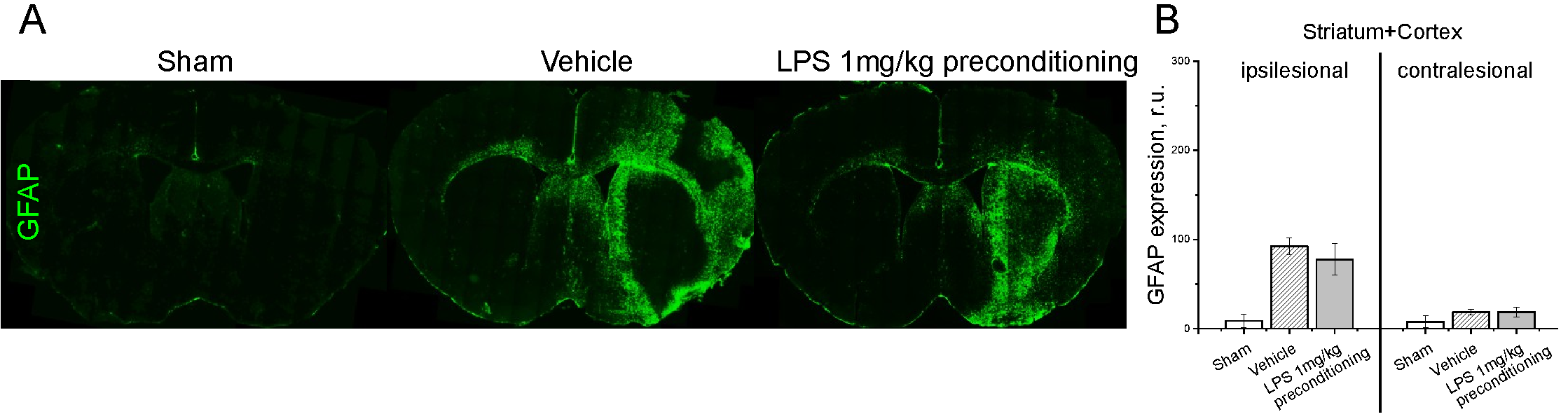

Supplement: FIGURE S3 — LPS pre-conditioning does not influence the post-ischemic astrogliosis. Panel (A) shows representative immunostainings of GFAP+ reactive astrocytes. GFAP immunoreactivity was quantified (B) in the entire ipsilesional and contralesional hemispheres (striatum plus cortex) of mice exposed to sham surgery or transient intraluminal MCAO. Vehicle or LPS (1 mg/kg) was intraperitoneally administered at 24 h prior to MCAO. No differences were noted between the groups. Data are means ± SD values (n = 4 animals/group). Scale bars, 1 mm. [file Image_3.tiff]

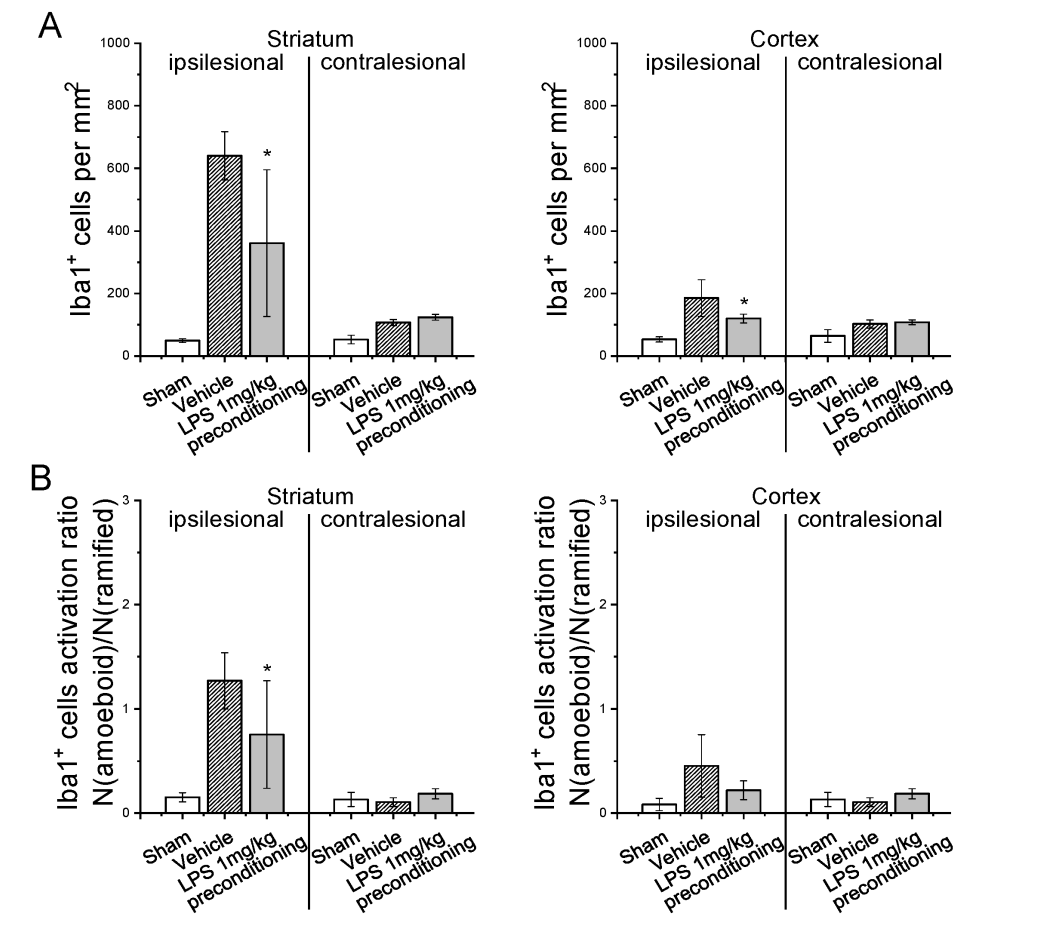

Supplement: FIGURE S4 — LPS preconditioning attenuates post-ischemic microglia/macrophage activation. The density of Iba1+ microglia/macrophages (A) and Iba1+ cell activation index (B) were quantified in striatum and cortex of mice exposed to sham surgery or transient intraluminal MCAO. Vehicle or LPS (1 mg/kg) was intraperitoneally administered at 24 h prior to MCAO. Data are means ± SD values. *p < 0.05 compared with vehicle group (n = 4 animals/group). Scale bars, 10 μm. [file Image_4.tiff]
